# On the Jensen–Shannon Symmetrization of Distances Relying on Abstract Means

**DOI:** 10.3390/e21050485

**Published:** 2019-05-11

**Authors:** Frank Nielsen

**Affiliations:** Sony Computer Science Laboratories, Takanawa Muse Bldg., 3-14-13, Higashigotanda, Shinagawa-ku, Tokyo 141-0022, Japan; Frank.Nielsen@acm.org or

**Keywords:** Jensen–Shannon divergence, Jeffreys divergence, resistor average distance, Bhattacharyya distance, *f*-divergence, Jensen/Burbea–Rao divergence, Bregman divergence, abstract weighted mean, quasi-arithmetic mean, mixture family, statistical *M*-mixture, exponential family, Gaussian family, Cauchy scale family, clustering

## Abstract

The Jensen–Shannon divergence is a renowned bounded symmetrization of the unbounded Kullback–Leibler divergence which measures the total Kullback–Leibler divergence to the average mixture distribution. However, the Jensen–Shannon divergence between Gaussian distributions is not available in closed form. To bypass this problem, we present a generalization of the Jensen–Shannon (JS) divergence using abstract means which yields closed-form expressions when the mean is chosen according to the parametric family of distributions. More generally, we define the JS-symmetrizations of any distance using parameter mixtures derived from abstract means. In particular, we first show that the geometric mean is well-suited for exponential families, and report two closed-form formula for (i) the geometric Jensen–Shannon divergence between probability densities of the same exponential family; and (ii) the geometric JS-symmetrization of the reverse Kullback–Leibler divergence between probability densities of the same exponential family. As a second illustrating example, we show that the harmonic mean is well-suited for the scale Cauchy distributions, and report a closed-form formula for the harmonic Jensen–Shannon divergence between scale Cauchy distributions. Applications to clustering with respect to these novel Jensen–Shannon divergences are touched upon.

## 1. Introduction and Motivations

### 1.1. Kullback–Leibler Divergence and Its Symmetrizations

Let (X,A) be a measurable space [1] where X denotes the sample space and A the σ-algebra of measurable events. Consider a positive measure μ (usually the Lebesgue measure $\mu_{L}$ with Borel σ-algebra $B(R^{d})$ or the counting measure $\mu_{c}$ with power set σ-algebra $2^{X}$). Denote by P the set of probability distributions.

The Kullback–Leibler Divergence [2] (KLD) KL:P×P→[0,∞] is the most fundamental distance [2] between probability distributions, defined by:

(1) $$\mathrm{KL}\left(P:Q\right):=\int p\log\frac{p}{q}d\mu ,$$

where *p* and *q* denote the Radon–Nikodym derivatives of probability measures *P* and *Q* with respect to μ (with P,Q≪μ). The KLD expression between *P* and *Q* in Equation (1) is independent of the dominating measure μ. Table A1 summarizes the various distances and their notations used in this paper.

The KLD is also called the relative entropy [2] because it can be written as the difference of the cross-entropy minus the entropy:

(2) $$\mathrm{KL}\left(p:q\right)=h_{\times }\left(p:q\right)-h\left(p\right),$$

where $h_{\times }$ denotes the cross-entropy [2]:

(3) $$h_{\times }\left(p:q\right):=\int p\log\frac{1}{q}d\mu ,$$

and

(4) $$h\left(p\right):=\int p\log\frac{1}{p}d\mu =h_{\times }\left(p:p\right),$$

denotes the *Shannon entropy* [2]. Although the formula of the Shannon entropy in Equation (4) unifies both the discrete case and the continuous case of probability distributions, the behavior of entropy in the discrete case and the continuous case is very different: When $\mu =\mu_{c}$, Equation (4) yields the discrete Shannon entropy which is always positive and upper bounded by log|X|. When $\mu =\mu_{L}$, Equation (4) defines the Shannon *differential entropy* which may be negative and unbounded [2] (e.g., the differential entropy of the Gaussian distribution N(m,σ) is $\frac{1}{2}\log\left(2\pi e\sigma^{2}\right)$). See also [3] for further important differences between the discrete case and the continuous case.

In general, the KLD is an asymmetric distance (i.e., KL(p:q)≠KL(q:p), hence the argument separator notation using the delimiter ‘:’) In information theory [2], it is customary to use the double bar notation ‘‖’ instead of the comma ‘,’ notation to avoid confusion with joint random variables. The *reverse KL divergence* or *dual KL divergence* is:

(5) $${\mathrm{KL}}^{*}\left(P:Q\right):=\mathrm{KL}\left(Q:P\right)=\int q\log\frac{q}{p}d\mu .$$

In general, the *reverse distance* or *dual distance* for a distance *D* is written as:

(6) $$D^{*}\left(p:q\right):=D\left(q:p\right).$$

One way to symmetrize the KLD is to consider the *Jeffreys Divergence* [4] (JD, Sir Harold Jeffreys (1891–1989) was a British statistician.):

(7) $$J\left(p;q\right):=\mathrm{KL}\left(p:q\right)+\mathrm{KL}\left(q:p\right)=\int \left(p-q\right)\log\frac{p}{q}d\mu =J\left(q;p\right).$$

However, this symmetric distance is not upper bounded, and its sensitivity can raise numerical issues in applications. Here, we used the optional argument separator notation ‘;’ to emphasize that the distance is symmetric but not necessarily a metric distance. This notation matches the notational convention of the mutual information if two joint random variables in information theory [2].

The symmetrization of the KLD may also be obtained using the harmonic mean instead of the arithmetic mean, yielding the *resistor average distance* [5] R(p;q):

(8) $$\begin{array}{ccc} \frac{1}{R(p;q)} & = & \frac{1}{2}\left(\frac{1}{\mathrm{KL}(p:q)}+\frac{1}{\mathrm{KL}(q:p)}\right), \end{array}$$

(9) $$\begin{array}{ccc} R(p;q) & = & \frac{2\left(\mathrm{KL}(p:q)+\mathrm{KL}(q:p)\right)}{\mathrm{KL}(p:q)\mathrm{KL}(q:p)}=\frac{2J(p;q)}{\mathrm{KL}(p:q)\mathrm{KL}(q:p)}. \end{array}$$

Another famous symmetrization of the KLD is the *Jensen–Shannon Divergence* [6] (JSD) defined by:

(10) $$\begin{array}{ccc} \mathrm{JS}(p;q) & := & \frac{1}{2}\left(\mathrm{KL}\left(p:\frac{p+q}{2}\right)+\mathrm{KL}\left(q:\frac{p+q}{2}\right)\right), \end{array}$$

(11) $$\begin{array}{cc} = & \frac{1}{2}\int \left(p\log\frac{2p}{p+q}+q\log\frac{2q}{p+q}\right)d\mu . \end{array}$$

This distance can be interpreted as the *total divergence to the average distribution* (see Equation (10)). The JSD can be rewritten as a *Jensen divergence* (or Burbea–Rao divergence [7]) for the negentropy generator −h (called Shannon information):

(12) $$\mathrm{JS}\left(p;q\right)=h\left(\frac{p+q}{2}\right)-\frac{h(p)+h(q)}{2}.$$

An important property of the Jensen–Shannon divergence compared to the Jeffreys divergence is that this distance is *always* bounded:

(13) 0≤JS(p:q)≤log2.

This follows from the fact that

(14) $$\mathrm{KL}\left(p:\frac{p+q}{2}\right)=\int p\log\frac{2p}{p+q}d\mu \leq \int p\log\frac{2p}{p}d\mu =\log2.$$

Finally, the square root of the JSD (i.e., $\sqrt{\mathrm{JS}}$) yields a *metric distance* satisfying the triangular inequality [8,9]. The JSD has found applications in many fields such as bioinformatics [10] and social sciences [11], just to name a few. Recently, the JSD has gained attention in the deep learning community with the *Generative Adversarial Networks* (GANs) [12]. In computer vision and pattern recognition, one often relies on information-theoretic techniques to perform registration and recognition tasks. For example, in [13], the authors use a mixture of Principal Axes Registrations (mPAR) whose parameters are estimated by minimizing the KLD between the considered two-point distributions. In [14], the authors parameterize both shapes and deformations using Gaussian Mixture Models (GMMs) to perform non-rigid shape registration. The lack of closed-form formula for the KLD between GMMs [15] spurred the use of other statistical distances which admit a closed-form expression for GMMs. For example, in [16], shape registration is performed by using the Jensen-Rényi divergence between GMMs. See also [17] for other information-theoretic divergences that admit closed-form formula for some statistical mixtures extending GMMs.

In information geometry [18], the KLD, JD and JSD are *invariant divergences* which satisfy the property of information monotonicity [18]. The class of (separable) distances satisfying the information monotonicity are exhaustively characterized as Csiszár’s *f*-divergences [19]. A *f-divergence* is defined for a convex generator function *f* strictly convex at 1 (with $f\left(1\right)=f^{\prime }\left(1\right)=0$) by:

(15) $$I_{f}\left(p:q\right)=\int pf\left(\frac{q}{p}\right)d\mu .$$

The Jeffreys and Jensen–Shannon *f*-generators are:

(16) $$\begin{array}{ccc} f_{J}\left(u\right) & := & (u-1)\log u, \end{array}$$

(17) $$\begin{array}{ccc} f_{\mathrm{JS}}\left(u\right) & := & -\left(u+1\right)\log\frac{1+u}{2}+u\log u. \end{array}$$

### 1.2. Statistical Distances and Parameter Divergences

In information and probability theory, the term “divergence” informally means a *statistical distance* [2]. However in information geometry [18], a divergence has a stricter meaning of being a smooth *parametric* distance (called a contrast function in [20]) from which a dual geometric structure can be derived [21,22].

Consider parametric distributions $p_{\theta }$ belonging to a parametric family of distributions $\left\{p_{\theta }\;\;:\;\;\theta \in \Theta \right\}$ (e.g., Gaussian family or Cauchy family), where Θ denotes the parameter space. Then a statistical distance *D* between distributions $p_{\theta }$ and $p_{\theta^{\prime }}$ amount to an equivalent *parameter distance*:

(18) $$P\left(\theta :\theta^{\prime }\right):=D\left(p_{\theta }:p_{\theta^{\prime }}\right).$$

For example, the KLD between two distributions belonging to the same exponential family (e.g., Gaussian family) amount to a reverse Bregman divergence for the cumulant generator *F* of the exponential family [23]:

(19) $$\mathrm{KL}\left(p_{\theta }:p_{\theta^{\prime }}\right)=B_{F}^{*}\left(\theta :\theta^{\prime }\right)=B_{F}\left(\theta^{\prime }:\theta \right).$$

A *Bregman divergence* $B_{F}$ is defined for a strictly convex and differentiable generator *F* as:

(20) $$B_{F}\left(\theta :\theta^{\prime }\right):=F\left(\theta \right)-F\left(\theta^{\prime }\right)-\langle \theta -\theta^{\prime },\nabla F\left(\theta^{\prime }\right)\rangle ,$$

where 〈·,·〉 is an inner product (usually the Euclidean dot product for vector parameters).

Similar to the interpretation of the Jensen–Shannon divergence (statistical divergence) as a Jensen divergence for the negentropy generator, the *Jensen–Bregman divergence* [7] ${\mathrm{JB}}_{F}$ (parametric divergence JBD) amounts to a Jensen divergence $J_{F}$ for a strictly convex generator F:Θ→R:

(21) $$\begin{array}{ccc} {\mathrm{JB}}_{F}\left(\theta :\theta^{\prime }\right) & := & \frac{1}{2}\left(B_{F}\left(\theta :\frac{\theta +\theta^{\prime }}{2}\right)+B_{F}\left(\theta^{\prime }:\frac{\theta +\theta^{\prime }}{2}\right)\right), \end{array}$$

(22) $$\begin{array}{cc} = & \frac{F\left(\theta \right)+F\left(\theta^{\prime }\right)}{2}-F\left(\frac{\theta +\theta^{\prime }}{2}\right)=:J_{F}\left(\theta :\theta^{\prime }\right), \end{array}$$

Let us introduce the notation ${\left(\theta_{p}\theta_{q}\right)}_{\alpha }:=\left(1-\alpha \right)\theta_{p}+\alpha \theta_{q}$ to denote the *linear interpolation* (LERP) of the parameters. Then we have more generally that the skew Jensen–Bregman divergence ${\mathrm{JB}}_{F}^{\alpha }\left(\theta :\theta^{\prime }\right)$ amounts to a skew Jensen divergence $J_{F}^{\alpha }\left(\theta :\theta^{\prime }\right)$:

(23) $$\begin{array}{ccc} {\mathrm{JB}}_{F}^{\alpha }\left(\theta :\theta^{\prime }\right) & := & \left(1-\alpha \right)B_{F}\left(\theta :{\left(\theta \theta^{\prime }\right)}_{\alpha }\right)+\alpha B_{F}\left(\theta^{\prime }:{\left(\theta \theta^{\prime }\right)}_{\alpha })\right), \end{array}$$

(24) $$\begin{array}{ccc} & = & {\left(F\left(\theta \right)F\left(\theta^{\prime }\right)\right)}_{\alpha }-F\left({\left(\theta \theta^{\prime }\right)}_{\alpha }\right)=:J_{F}^{\alpha }\left(\theta :\theta^{\prime }\right), \end{array}$$

### 1.3. J-Symmetrization and JS-Symmetrization of Distances

For any arbitrary distance D(p:q), we can define its *skew J-symmetrization* for α∈[0,1] by:

(25) $$J_{D}^{\alpha }\left(p:q\right):=\left(1-\alpha \right)D\left(p:q\right)+\alpha D\left(q:p\right),$$

and its *JS-symmetrization* by:

(26) $$\begin{array}{ccc} {\mathrm{JS}}_{D}^{\alpha }\left(p:q\right) & := & \left(1-\alpha \right)D\left(p:(1-\alpha )p+\alpha q\right)+\alpha D\left(q:(1-\alpha )p+\alpha q\right), \end{array}$$

(27) $$\begin{array}{cc} = & \left(1-\alpha \right)D\left(p:{\left(pq\right)}_{\alpha }\right)+\alpha D\left(q:{\left(pq\right)}_{\alpha }\right). \end{array}$$

Usually, $\alpha =\frac{1}{2}$, and for notational brevity, we drop the superscript: ${\mathrm{JS}}_{D}\left(p:q\right):={\mathrm{JS}}_{D}^{\frac{1}{2}}\left(p:q\right)$. The Jeffreys divergence is twice the *J*-symmetrization of the KLD, and the Jensen–Shannon divergence is the JS-symmetrization of the KLD.

The *J*-symmetrization of a *f*-divergence $I_{f}$ is obtained by taking the generator

(28) $$f_{\alpha }^{J}\left(u\right)=\left(1-\alpha \right)f\left(u\right)+\alpha f^{⋄}\left(u\right),$$

where $f^{⋄}\left(u\right)=uf\left(\frac{1}{u}\right)$ is the *conjugate* generator:

(29) $$I_{f^{⋄}}\left(p:q\right)=I_{f}^{*}\left(p:q\right)=I_{f}\left(q:p\right).$$

The JS-symmetrization of a *f*-divergence

(30) $$I_{f}^{\alpha }\left(p:q\right):=\left(1-\alpha \right)I_{f}\left(p:{\left(pq\right)}_{\alpha }\right)+\alpha I_{f}\left(q:{\left(pq\right)}_{\alpha }\right),$$

with ${\left(pq\right)}_{\alpha }=\left(1-\alpha \right)p+\alpha q$ is obtained by taking the generator

(31) $$f_{\alpha }^{\mathrm{JS}}\left(u\right):=\left(1-\alpha \right)f\left(\alpha u+1-\alpha \right)+\alpha f\left(\alpha +\frac{1-\alpha }{u}\right).$$

We check that we have:

(32) $$I_{f}^{\alpha }\left(p:q\right)=\left(1-\alpha \right)I_{f}\left(p:{\left(pq\right)}_{\alpha }\right)+\alpha I_{f}\left(q:{\left(pq\right)}_{\alpha }\right)=I_{f}^{1-\alpha }\left(q:p\right)=I_{f_{\alpha }^{\mathrm{JS}}}\left(p:q\right).$$

A family of symmetric distances unifying the Jeffreys divergence with the Jensen–Shannon divergence was proposed in [24]. Finally, let us mention that once we have symmetrized a distance *D*, we may also metrize this symmetric distance by choosing (when it exists) the largest exponent δ>0 such that $D^{\delta }$ becomes a metric distance [8,25,26,27,28].

### 1.4. Contributions and Paper Outline

The paper is organized as follows:

Section 2 reports the special case of mixture families in information geometry [18] for which the Jensen–Shannon divergence can be expressed as a Bregman divergence (Theorem 1), and highlight the lack of closed-form formula when considering exponential families. This fact precisely motivated this work.

Section 3 introduces the generalized Jensen–Shannon divergences using statistical mixtures derived from abstract weighted means (Definitions 2 and 5), presents the JS-symmetrization of statistical distances, and report a sufficient condition to get bounded JS-symmetrizations (Property 1).

In Section 4.1, we consider the calculation of the geometric JSD between members of the same exponential family (Theorem 2) and instantiate the formula for the multivariate Gaussian distributions (Corollary 1). We discuss about applications for *k*-means clustering in Section 4.1.2. In Section 4.2, we illustrate the method with another example that calculates in closed form the harmonic JSD between scale Cauchy distributions (Theorem 4).

Finally, we wrap up and conclude this work in Section 5.

## 2. Jensen–Shannon Divergence in Mixture and Exponential Families

We are interested to calculate the JSD between densities belonging to parametric families of distributions.

A trivial example is when $p=(p_{0},\ldots ,p_{D})$ and $q=(q_{0},\ldots ,q_{D})$ are categorical distributions: The average distribution $\frac{p+q}{2}$ is a again categorical distribution, and the JSD is expressed plainly as:

(33) $$\mathrm{JS}\left(p,q\right)=\frac{1}{2}\sum_{i=0}^{D}\left(p_{i}\log\frac{2p_{i}}{p_{i}+q_{i}}+q_{i}\log\frac{2q_{i}}{p_{i}+q_{i}}\right).$$

Another example is when $p=m_{\theta_{p}}$ and $q=m_{\theta_{q}}$ both belong to the same *mixture family* [18] M:

(34) $$M:=\left\{m_{\theta }\left(x\right)=\left(1-\sum_{i=1}^{D}\theta_{i}p_{i}\left(x\right)\right)p_{0}\left(x\right)+\sum_{i=1}^{D}\theta_{i}p_{i}\left(x\right)\;:\;\theta_{i}>0,\sum_{i}\theta_{i}<1\right\},$$

for linearly independent component distributions $p_{0},p_{1},\ldots ,p_{D}$. We have [29]:

(35) $$\mathrm{KL}\left(m_{\theta_{p}}:m_{\theta_{q}}\right)=B_{F}\left(\theta_{p}:\theta_{q}\right),$$

where $B_{F}$ is a Bregman divergence defined in Equation (20) obtained for the convex negentropy generator [29] $F\left(\theta \right)=-h\left(m_{\theta }\right)$. The proof that F(θ) is a strictly convex function is not trivial [30].

The mixture families include the family of categorical distributions over a finite alphabet $X\;=\;\{E_{0},\ldots ,E_{D}\}$ (the *D*-dimensional probability simplex) since those categorical distributions form a mixture family with $p_{i}\left(x\right):=\mathrm{Pr}\left(X=E_{i}\right)=\delta_{E_{i}}\left(x\right)$. Beware that mixture families impose to prescribe the component distributions. Therefore, a density of a mixture family is a special case of statistical mixtures (e.g., GMMs) with prescribed component distributions.

The mathematical identity of Equation (35) that does not yield a practical formula since F(θ) is usually not itself available in closed form. Worse, the Bregman generator can be non-analytic [31]. Nevertheless, this identity is useful for computing the right-sided Bregman centroid (left KL centroid of mixtures) since this centroid is equivalent to the center of mass, and independent of the Bregman generator [29].

Since the mixture of mixtures is also a mixture, specifically

(36) $$\frac{m_{\theta_{p}}+m_{\theta_{q}}}{2}=m_{\frac{\theta_{p}+\theta_{q}}{2}}\in M,$$

it follows that we get a closed-form expression for the JSD between mixtures belonging to M.

**Theorem 1** (JSD between mixtures)**.** *The Jensen–Shannon divergence between two distributions $p=m_{\theta_{p}}$ and $q=m_{\theta_{q}}$ belonging to the same mixture family M is expressed as a Jensen–Bregman divergence for the negentropy generator F:*

(37) $$\mathrm{JS}\left(m_{\theta_{p}},m_{\theta_{q}}\right)=\frac{1}{2}\left(B_{F}\left(\theta_{p}:\frac{\theta_{p}+\theta_{q}}{2}\right)+B_{F}\left(\theta_{q}:\frac{\theta_{p}+\theta_{q}}{2}\right)\right).$$

*This amounts to calculate the Jensen divergence:*

(38) $$\mathrm{JS}\left(m_{\theta_{p}},m_{\theta_{q}}\right)=J_{F}\left(\theta_{1};\theta_{2}\right)={\left(F\left(\theta_{1}\right)F\left(\theta_{2}\right)\right)}_{\frac{1}{2}}-F\left({\left(\theta_{1}\theta_{2}\right)}_{\frac{1}{2}}\right),$$

*where ${\left(v_{1}v_{2}\right)}_{\alpha }:=\left(1-\alpha \right)v_{1}+\alpha v_{2}$.*

Now, consider distributions $p=e_{\theta_{p}}$ and $q=e_{\theta_{q}}$ belonging to the same *exponential family* [18] E:

(39) $$E:=\left\{e_{\theta }\left(x\right)=\exp\left(\theta^{⊤}x-F\left(\theta \right)\right)\;:\;\theta \in \Theta \right\},$$

where

(40) $$\Theta :=\left\{\theta \in R^{D}\;:\;\int \exp\left(\theta^{⊤}x\right)d\mu <\infty \right\},$$

denotes the natural parameter space. We have [18]:

(41) $$\mathrm{KL}\left(e_{\theta_{p}}:e_{\theta_{q}}\right)=B_{F}\left(\theta_{q}:\theta_{p}\right),$$

where *F* denotes the log-normalizer or cumulant function of the exponential family [18].

However, $\frac{e_{\theta_{p}}+e_{\theta_{q}}}{2}$*does not* belong to E in general, except for the case of the categorical/multinomial family which is both an exponential family and a mixture family [18].

For example, the mixture of two Gaussian distributions with distinct components is *not* a Gaussian distribution. Thus, it is not obvious to get a closed-form expression for the JSD in that case. This limitation precisely motivated the introduction of generalized JSDs defined in the next section.

Notice that in [32,33], it is shown how to express or approximate the *f*-divergences using expansions of power χ pseudo-distances. These power chi distances can all be expressed in closed form when dealing with isotropic Gaussians. This result holds for the JSD since the JSD is a *f*-divergence [33].

## 3. Generalized Jensen–Shannon Divergences

We first define abstract means *M*, and then generic statistical *M*-mixtures from which generalized Jensen–Shannon divergences are built thereof.

### Definitions

Consider an *abstract mean* [34] *M*. That is, a continuous bivariate function M(·,·):I×I→I on an interval I⊂R that satisfies the following *in-betweenness* property:

(42) inf{x,y}≤M(x,y)≤sup{x,y},∀x,y∈I.

Using the unique *dyadic expansion* of real numbers, we can always build a corresponding *weighted mean* $M_{\alpha }\left(p,q\right)$ (with α∈[0,1]) following the construction reported in [34] (page 3) such that $M_{0}\left(p,q\right)=p$ and $M_{1}\left(p,q\right)=q$. In the remainder, we consider I=(0,∞).

Examples of common weighted means are:

- the *arithmetic mean* $A_{\alpha }\left(x,y\right)=\left(1-\alpha \right)x+\alpha_{y}$,
- the *geometric mean* $G_{\alpha }\left(x,y\right)=x^{1-\alpha }y^{\alpha }$, and
- the *harmonic mean* $H_{\alpha }\left(x,y\right)=\frac{xy}{(1-\alpha )y+\alpha x}$.

These means can be unified using the concept of *quasi-arithmetic means* [34] (also called Kolmogorov–Nagumo means):

(43) $$M_{\alpha }^{h}\left(x,y\right):=h^{-1}\left((1-\alpha )h(x)+\alpha h(y)\right),$$

where *h* is a strictly monotonous function. For example, the geometric mean $G_{\alpha }\left(x,y\right)$ is obtained as $M_{\alpha }^{h}\left(x,y\right)$ for the generator h(u)=log(u). Rényi used the concept of quasi-arithmetic means instead of the arithmetic mean to define axiomatically the Rényi entropy [35] of order α in information theory [2].

For any abstract weighted mean, we can build a statistical mixture called a *M*-mixture as follows:

**Definition 1** (*M*-mixture)**.** *The*$M_{\alpha }$-interpolation *${\left(pq\right)}_{\alpha }^{M}$ (with α∈[0,1]) of densities p and q with respect to a mean M is a α-weighted M-mixture defined by:*

(44) $${\left(pq\right)}_{\alpha }^{M}\left(x\right):=\frac{M_{\alpha }\left(p\left(x\right),q\left(x\right)\right)}{Z_{\alpha }^{M}\left(p:q\right)},$$

*where*

(45) $$Z_{\alpha }^{M}\left(p:q\right)=\int_{t\in X}M_{\alpha }\left(p\left(t\right),q\left(t\right)\right)d\mu \left(t\right)=:\langle M_{\alpha }\left(p,q\right)\rangle .$$

*is the normalizer function (or scaling factor) ensuring that ${\left(pq\right)}_{\alpha }^{M}\in P$. (The bracket notation $\langle f\rangle$ denotes the integral of f over X.)*

The *A*-mixture ${\left(pq\right)}_{\alpha }^{A}\left(x\right)=\left(1-\alpha \right)p\left(x\right)+\alpha q\left(x\right)$ (‘A’ standing for the arithmetic mean) represents the usual statistical mixture [36] (with $Z_{\alpha }^{A}\left(p:q\right)=1$). The *G*-mixture ${\left(pq\right)}_{\alpha }^{G}\left(x\right)=\frac{p{\left(x\right)}^{1-\alpha }q{\left(x\right)}^{\alpha }}{Z_{\alpha }^{G}\left(p:q\right)}$ of two distributions p(x) and q(x) (’G’ standing for the geometric mean *G*) is an exponential family of order [37] 1:

(46) $${\left(pq\right)}_{\alpha }^{G}\left(x\right)=\exp\left(\left(1-\alpha \right)p\left(x\right)+\alpha q\left(x\right)-\log Z_{\alpha }^{G}\left(p:q\right)\right).$$

The two-component *M*-mixture can be generalized to a *k*-component *M*-mixture with $\alpha \in \Delta_{k-1}$, the (k−1)-dimensional standard simplex:

(47) $${\left(p_{1}\ldots p_{k}\right)}_{\alpha }^{M}:=\frac{p_{1}{\left(x\right)}^{\alpha_{1}}\times \ldots \times p_{k}{\left(x\right)}^{\alpha_{k}}}{Z_{\alpha }\left(p_{1},\ldots ,p_{k}\right)},$$

where $Z_{\alpha }\left(p_{1},\ldots ,p_{k}\right):=\int_{X}p_{1}{\left(x\right)}^{\alpha_{1}}\times \ldots \times p_{k}{\left(x\right)}^{\alpha_{k}}d\mu \left(x\right)$.

For a given pair of distributions *p* and *q*, the set $\left\{M_{\alpha }\left(p\left(x\right),q\left(x\right)\right)\;:\;\alpha \in \left[0,1\right]\right\}$ describes a path in the space of probability density functions. This density interpolation scheme was investigated for quasi-arithmetic weighted means in [38,39,40]. In [41], the authors study the Fisher information matrix for the α-mixture models (using α-power means).

We call ${\left(pq\right)}_{\alpha }^{M}$ the α-weighted *M*-mixture, thus extending the notion of α-mixtures [42] obtained for power means $P_{\alpha }$. Notice that abstract means have also been used to generalize Bregman divergences using the concept of *(M,N)-convexity* [43].

Let us state a first generalization of the Jensen–Shannon divergence:

**Definition 2** (*M*-Jensen–Shannon divergence)**.** *For a mean M, the skew M-Jensen–Shannon divergence (for α∈[0,1]) is defined by*

(48) $${\mathrm{JS}}^{M_{\alpha }}\left(p:q\right):=\left(1-\alpha \right)\mathrm{KL}\left(p:{\left(pq\right)}_{\alpha }^{M}\right)+\alpha \mathrm{KL}\left(q:{\left(pq\right)}_{\alpha }^{M}\right)$$

When $M_{\alpha }=A_{\alpha }$, we recover the ordinary Jensen–Shannon divergence since $A_{\alpha }\left(p:q\right)={\left(pq\right)}_{\alpha }$ (and $Z_{\alpha }^{A}\left(p:q\right)=1$).

We can extend the definition to the JS-symmetrization of any distance:

**Definition 3** (*M*-JS symmetrization)**.** *For a mean M and a distance D, the skew M-JS symmetrization of D (for α∈[0,1]) is defined by*

(49) $${\mathrm{JS}}_{D}^{M_{\alpha }}\left(p:q\right):=\left(1-\alpha \right)D\left(p:{\left(pq\right)}_{\alpha }^{M}\right)+\alpha D\left(q:{\left(pq\right)}_{\alpha }^{M}\right)$$

By notation, we have ${\mathrm{JS}}^{M_{\alpha }}\left(p:q\right)={\mathrm{JS}}_{\mathrm{KL}}^{M_{\alpha }}\left(p:q\right)$. That is, the arithmetic JS-symmetrization of the KLD is the JSD.

Let us define the α-skew *K*-divergence [6,44] $K_{\alpha }\left(p:q\right)$ as

(50) $$K_{\alpha }\left(p:q\right):=\mathrm{KL}\left(p:\left(1-\alpha \right)p+\alpha q\right)=\mathrm{KL}\left(p:{\left(pq\right)}_{\alpha }\right),$$

where ${\left(pq\right)}_{\alpha }\left(x\right):=\left(1-\alpha \right)p\left(x\right)+\alpha q\left(x\right)$. Then the Jensen–Shannon divergence and the Jeffreys divergence can be rewritten [24] as

(51) $$\begin{array}{ccc} \mathrm{JS}\left(p;q\right) & = & \frac{1}{2}\left(K_{\frac{1}{2}}\left(p:q\right)+K_{\frac{1}{2}}\left(q:p\right)\right), \end{array}$$

(52) $$\begin{array}{ccc} J\left(p;q\right) & = & K_{1}\left(p:q\right)+K_{1}\left(q:p\right), \end{array}$$

since $\mathrm{KL}\left(p:q\right)=K_{1}\left(p:q\right)$. Then ${\mathrm{JS}}_{\alpha }\left(p:q\right)=\left(1-\alpha \right)K_{\alpha }\left(p:q\right)+\alpha K_{1-\alpha }\left(q:p\right)$. Similarly, we can define the generalized skew *K*-divergence:

(53) $$K_{D}^{M_{\alpha }}\left(p:q\right):=D\left(p:{\left(pq\right)}_{\alpha }^{M}\right).$$

The success of the JSD compared to the JD in applications is partially due to the fact that the JSD is upper bounded by log2. So, one question to ask is whether those generalized JSDs are upper bounded or not?

To report a sufficient condition, let us first introduce the dominance relationship between means: We say that a mean *M* dominates a mean *N* when M(x,y)≥N(x,y) for all x,y≥0, see [34]. In that case we write concisely M≥N. For example, the Arithmetic-Geometric-Harmonic (AGH) inequality states that A≥G≥H.

Consider the term

(54) $$\begin{array}{ccc} \mathrm{KL}(p:{\left(pq\right)}_{\alpha }^{M}) & = & \int p\left(x\right)\log\frac{p\left(x\right)Z_{\alpha }^{M}\left(p,q\right)}{M_{\alpha }\left(p\left(x\right),q\left(x\right)\right)}d\mu \left(x\right), \end{array}$$

(55) $$\begin{array}{cc} = & \log Z_{\alpha }^{M}\left(p,q\right)+\int p\left(x\right)\log\frac{p(x)}{M_{\alpha }\left(p\left(x\right),q\left(x\right)\right)}d\mu \left(x\right). \end{array}$$

When mean $M_{\alpha }$ dominates the arithmetic mean $A_{\alpha }$, we have

$$\int p\left(x\right)\log\frac{p(x)}{M_{\alpha }\left(p\left(x\right),q\left(x\right)\right)}d\mu \left(x\right)\leq \int p\left(x\right)\log\frac{p(x)}{A_{\alpha }\left(p\left(x\right),q\left(x\right)\right)}d\mu \left(x\right),$$

and

$$\int p\left(x\right)\log\frac{p(x)}{A_{\alpha }\left(p\left(x\right),q\left(x\right)\right)}d\mu \left(x\right)\leq \int p\left(x\right)\log\frac{p(x)}{\left(1-\alpha )p(x\right)}d\mu \left(x\right)=-\log\left(1-\alpha \right).$$

Notice that $Z_{\alpha }^{A}\left(p:q\right)=1$ (when M=A is the arithmetic mean), and we recover the fact that the α-skew Jensen–Shannon divergence is upper bounded by −log(1−α) (e.g., log2 when $\alpha =\frac{1}{2}$).

We summarize the result in the following property:

**Property** **1** (Upper bound on *M*-JSD)**.** *The M-JSD is upper bounded by $\log\frac{Z_{\alpha }^{M}\left(p,q\right)}{1-\alpha }$ when M≥A.*

Let us observe that dominance of means can be used to define distances: For example, the celebrated α-divergences

(56) $$I_{\alpha }\left(p:q\right)=\int \left(\alpha p\left(x\right)+\left(1-\alpha \right)q\left(x\right)-p{\left(x\right)}^{\alpha }q{\left(x\right)}^{1-\alpha }\right)d\mu \left(x\right),\;\alpha \notin \left\{0,1\right\}$$

can be interpreted as a difference of two means, the arithmetic mean and the geometry mean:

(57) $$I_{\alpha }\left(p:q\right)=\int \left(A_{\alpha }\left(q\left(x\right):p\left(x\right)\right)-G_{\alpha }\left(q\left(x\right):p\left(x\right)\right)\right)d\mu \left(x\right).$$

We can also define the generalized Jeffreys divergence as follows:

**Definition** **4** (*N*-Jeffreys divergence)**.** *For a mean N, the skew N-Jeffreys divergence (for β∈[0,1]) is defined by*

(58) $$\begin{array}{ccc} J^{N_{\beta }}\left(p:q\right) & := & N_{\beta }\left(\mathrm{KL}\left(p:q\right),\mathrm{KL}\left(q:p\right)\right). \end{array}$$

This definition includes the (scaled) *resistor average distance* [5] R(p;q), obtained for the *harmonic mean* N=H for the KLD with skew parameter $\beta =\frac{1}{2}$:

(59) $$\begin{array}{ccc} \frac{1}{R(p;q)} & = & \frac{1}{2}\left(\frac{1}{\mathrm{KL}(p:q)}+\frac{1}{\mathrm{KL}(q:p)}\right), \end{array}$$

(60) $$\begin{array}{ccc} R(p;q) & = & \frac{2J(p;q)}{\mathrm{KL}(p:q)\mathrm{KL}(q:p)}. \end{array}$$

In [5], the factor $\frac{1}{2}$ is omitted to keep the spirit of the original Jeffreys divergence.

We can further extend this definition for any arbitrary divergence *D* as follows:

**Definition 5** (Skew (M,N)-*D* divergence)**.** *The skew (M,N)-divergence with respect to weighted means $M_{\alpha }$ and $N_{\beta }$ as follows:*

(61) $${\mathrm{JS}}_{D}^{M_{\alpha },N_{\beta }}\left(p:q\right):=N_{\beta }\left(D\left(p:{\left(pq\right)}_{\alpha }^{M}\right),D\left(q:{\left(pq\right)}_{\alpha }^{M}\right)\right).$$

We now show how to choose the abstract mean according to the parametric family of distributions to obtain some closed-form formula for some statistical distances.

## 4. Some Closed-Form Formula for the *M*-Jensen–Shannon Divergences

Our motivation to introduce these novel families of *M*-Jensen–Shannon divergences is to obtain closed-form formula when probability densities belong to some given parametric families $P_{\Theta }$. We shall illustrate the principle of the method to choose the right abstract mean for the considered parametric family, and report corresponding formula for the following two case studies:

1. The *geometric G-Jensen–Shannon divergence* for the *exponential families* (Section 4.1), and
2. the *harmonic H-Jensen–Shannon divergence* for the family of *Cauchy scale distributions* (Section 4.2).

Recall that the arithmetic *A*-Jensen–Shannon divergence is well-suited for mixture families (Theorem 1).

### 4.1. The Geometric G-Jensen–Shannon Divergence

Consider an exponential family [37] $E_{F}$ with log-normalizer *F*:

(62) $$E_{F}=\left\{p_{\theta }\left(x\right)d\mu =\exp\left(\theta^{⊤}x-F\left(\theta \right)\right)d\mu \;:\;\theta \in \Theta \right\},$$

and natural parameter space

(63) $$\Theta =\left\{\theta \;:\;\int_{X}\exp\left(\theta^{⊤}x\right)d\mu <\infty \right\}.$$

The log-normalizer (a log-Laplace function also called log-partition or cumulant function) is a real analytic convex function.

We seek for a mean *M* such that the weighted *M*-mixture density ${\left(p_{\theta_{1}}p_{\theta_{2}}\right)}_{\alpha }^{M}$ of two densities $p_{\theta_{1}}$ and $p_{\theta_{2}}$ of the same exponential family yields another density of that exponential family (e.g., $p_{{\left(\theta_{1}\theta_{2}\right)}_{\alpha }}$). When considering exponential families, choose the *weighted geometric mean* $G_{\alpha }$ for the abstract mean $M_{\alpha }\left(x,y\right)$: $M_{\alpha }\left(x,y\right)=G_{\alpha }\left(x,y\right)=x^{1-\alpha }y^{\alpha }$, for x,y>0. Indeed, it is well-known that the normalized weighted product of distributions belonging to the same exponential family also belongs to this exponential family [45]:

(64) $$\begin{array}{ccc} \forall x\in X,\;{\left(p_{\theta_{1}}p_{\theta_{2}}\right)}_{\alpha }^{G}\left(x\right) & := & \frac{G_{\alpha }\left(p_{\theta_{1}}\left(x\right),p_{\theta_{2}}\left(x\right)\right)}{\int G_{\alpha }\left(p_{\theta_{1}}\left(t\right),p_{\theta_{2}}\left(t\right)\right)d\mu \left(t\right)}=\frac{p_{\theta_{1}}^{1-\alpha }\left(x\right)p_{\theta_{2}}^{\alpha }\left(x\right)}{Z_{\alpha }^{G}\left(p:q\right)}, \end{array}$$

(65) $$\begin{array}{cc} = & p_{{\left(\theta_{1}\theta_{2}\right)}_{\alpha }}\left(x\right), \end{array}$$

where the normalization factor is

(66) $$Z_{\alpha }^{G}\left(p:q\right)=\exp\left(-J_{F}^{\alpha }\left(\theta_{1}:\theta_{2}\right)\right),$$

for the skew Jensen divergence $J_{F}^{\alpha }$ defined by:

(67) $$J_{F}^{\alpha }\left(\theta_{1}:\theta_{2}\right):={\left(F\left(\theta_{1}\right)F\left(\theta_{2}\right)\right)}_{\alpha }-F\left({\left(\theta_{1}\theta_{2}\right)}_{\alpha }\right).$$

Notice that since the natural parameter space Θ is convex, the distribution $p_{{\left(\theta_{1}\theta_{2}\right)}_{\alpha }}\in E_{F}$ (since ${\left(\theta_{1}\theta_{2}\right)}_{\alpha }\in \Theta$).

Thus, it follows that we have:

(68) $$\begin{array}{ccc} \mathrm{KL}\left(p_{\theta }:{\left(p_{\theta_{1}}p_{\theta_{2}}\right)}_{\alpha }^{G}\right) & = & \mathrm{KL}\left(p_{\theta }:p_{{\left(\theta_{1}\theta_{2}\right)}_{\alpha }}\right), \end{array}$$

(69) $$\begin{array}{cc} = & B_{F}\left({\left(\theta_{1}\theta_{2}\right)}_{\alpha }:\theta \right). \end{array}$$

This allows us to conclude that the *G-Jensen–Shannon divergence* admits the following closed-form expression between densities belonging to the same exponential family:

(70) $$\begin{array}{ccc} {\mathrm{JS}}_{\alpha }^{G}\left(p_{\theta_{1}}:p_{\theta_{2}}\right) & := & \left(1-\alpha \right)\mathrm{KL}\left(p_{\theta_{1}}:{\left(p_{\theta_{1}}p_{\theta_{2}}\right)}_{\alpha }^{G}\right)+\alpha \mathrm{KL}\left(p_{\theta_{2}}:{\left(p_{\theta_{1}}p_{\theta_{2}}\right)}_{\alpha }^{G}\right), \end{array}$$

(71) $$\begin{array}{cc} = & \left(1-\alpha \right)B_{F}\left({\left(\theta_{1}\theta_{2}\right)}_{\alpha }:\theta_{1}\right)+\alpha B_{F}\left({\left(\theta_{1}\theta_{2}\right)}_{\alpha }:\theta_{2}\right). \end{array}$$

Please note that since ${\left(\theta_{1}\theta_{2}\right)}_{\alpha }-\theta_{1}=\alpha \left(\theta_{2}-\theta_{1}\right)$ and ${\left(\theta_{1}\theta_{2}\right)}_{\alpha }-\theta_{2}=\left(1-\alpha \right)\left(\theta_{1}-\theta_{2}\right)$, it follows that $\left(1-\alpha \right)B_{F}\left(\theta_{1}:{\left(\theta_{1}\theta_{2}\right)}_{\alpha }\right)+\alpha B_{F}\left(\theta_{2}:{\left(\theta_{1}\theta_{2}\right)}_{\alpha }\right)=J_{F}^{\alpha }\left(\theta_{1}:\theta_{2}\right)$.

The *dual divergence* [46] $D^{*}$ (with respect to the reference argument) or *reverse divergence* of a divergence *D* is defined by swapping the calling arguments: $D^{*}\left(\theta :\theta^{\prime }\right):=D\left(\theta^{\prime }:\theta \right)$.

Thus, if we defined the Jensen–Shannon divergence for the dual KL divergence ${\mathrm{KL}}^{*}\left(p:q\right):=\mathrm{KL}\left(q:p\right)$

(72) $$\begin{array}{ccc} {\mathrm{JS}}_{{\mathrm{KL}}^{*}}\left(p:q\right) & := & \frac{1}{2}\left({\mathrm{KL}}^{*}\left(p:\frac{p+q}{2}\right)+{\mathrm{KL}}^{*}\left(q:\frac{p+q}{2}\right)\right), \end{array}$$

(73) $$\begin{array}{cc} = & \frac{1}{2}\left(\mathrm{KL}\left(\frac{p+q}{2}:p\right)+\mathrm{KL}\left(\frac{p+q}{2}:q\right)\right), \end{array}$$

then we obtain:

(74) $$\begin{array}{ccc} {\mathrm{JS}}_{{\mathrm{KL}}^{*}}^{G_{\alpha }}\left(p_{\theta_{1}}:p_{\theta_{2}}\right) & := & \left(1-\alpha \right)\mathrm{KL}\left({\left(p_{\theta_{1}}p_{\theta_{2}}\right)}_{\alpha }^{G}:p_{\theta_{1}}\right)+\alpha \mathrm{KL}\left({\left(p_{\theta_{1}}p_{\theta_{2}}\right)}_{\alpha }^{G}:p_{\theta_{2}}\right), \end{array}$$

(75) $$\begin{array}{cc} = & \left(1-\alpha \right)B_{F}\left(\theta_{1}:{\left(\theta_{1}\theta_{2}\right)}_{\alpha }\right)+\alpha B_{F}\left(\theta_{2}:{\left(\theta_{1}\theta_{2}\right)}_{\alpha }\right)={\mathrm{JB}}_{F}^{\alpha }\left(\theta_{1}:\theta_{2}\right), \end{array}$$

(76) $$\begin{array}{cc} = & \left(1-\alpha \right)F\left(\theta_{1}\right)+\alpha F\left(\theta_{2}\right)-F\left({\left(\theta_{1}\theta_{2}\right)}_{\alpha }\right), \end{array}$$

(77) $$\begin{array}{cc} = & J_{F}^{\alpha }\left(\theta_{1}:\theta_{2}\right). \end{array}$$

Please note that ${\mathrm{JS}}_{D^{*}}\neq {{\mathrm{JS}}_{D}}^{*}$.

In general, the JS-symmetrization for the reverse KL divergence is

(78) $$\begin{array}{ccc} {\mathrm{JS}}_{{\mathrm{KL}}^{*}}\left(p;q\right) & = & \frac{1}{2}\left(\mathrm{KL}\left(\frac{p+q}{2}:p\right)+\mathrm{KL}\left(\frac{p+q}{2}:q\right)\right), \end{array}$$

(79) $$\begin{array}{cc} = & \int m\log\frac{m}{\sqrt{pq}}d\mu =\int A\left(p,q\right)\log\frac{A(p,q)}{G(p,q)}d\mu , \end{array}$$

where $m=\frac{p+q}{2}=A\left(p,q\right)$ and $G\left(p,q\right)=\sqrt{pq}$. Since A≥G (arithmetic-geometric inequality), it follows that ${\mathrm{JS}}_{{\mathrm{KL}}^{*}}\left(p;q\right)\geq 0$.

**Theorem** **2** (*G*-JSD and its dual JS-symmetrization in exponential families)**.** *The α-skew G-Jensen–Shannon divergence ${\mathrm{JS}}^{G_{\alpha }}$ between two distributions $p_{\theta_{1}}$ and $p_{\theta_{2}}$ of the same exponential family $E_{F}$ is expressed in closed form for α∈(0,1) as:*

(80) $$\begin{array}{ccc} {\mathrm{JS}}^{G_{\alpha }}\left(p_{\theta_{1}}:p_{\theta_{2}}\right) & = & \left(1-\alpha \right)B_{F}\left({\left(\theta_{1}\theta_{2}\right)}_{\alpha }:\theta_{1}\right)+\alpha B_{F}\left({\left(\theta_{1}\theta_{2}\right)}_{\alpha }:\theta_{2}\right), \end{array}$$

(81) $$\begin{array}{ccc} {\mathrm{JS}}_{{\mathrm{KL}}^{*}}^{G_{\alpha }}\left(p_{\theta_{1}}:p_{\theta_{2}}\right) & = & {\mathrm{JB}}_{F}^{\alpha }\left(\theta_{1}:\theta_{2}\right)=J_{F}^{\alpha }\left(\theta_{1}:\theta_{2}\right). \end{array}$$

#### 4.1.1. Case Study: The Multivariate Gaussian Family

Consider the *exponential family* [18,37] of multivariate Gaussian distributions [47,48,49]

(82) $$\{N\left(\mu ,\Sigma \right)\;:\;\mu \in R^{d},\Sigma ≻0\}.$$

The multivariate Gaussian family is also called the *multivariate normal* family in the literature, or MVN family for short.

Let $\lambda :=\left(\lambda_{v},\lambda_{M}\right)=\left(\mu ,\Sigma \right)$ denote the *composite* (vector,matrix) parameter of an MVN. The *d*-dimensional MVN density is given by

(83) $$\begin{array}{ccc} p_{\lambda }\left(x;\lambda \right) & := & \frac{1}{{\left(2\pi \right)}^{\frac{d}{2}}\sqrt{|\lambda_{M}|}}\exp\left(-\frac{1}{2}{\left(x-\lambda_{v}\right)}^{⊤}\lambda_{M}^{-1}\left(x-\lambda_{v}\right)\right), \end{array}$$

where |·| denotes the matrix determinant. The natural parameters θ are also expressed using both a vector parameter $\theta_{v}$ and a matrix parameter $\theta_{M}$ in a compound object $\theta =(\theta_{v},\theta_{M})$. By defining the following *compound inner product* on a composite (vector,matrix) object

(84) $$\langle \theta ,\theta^{\prime }\rangle :=\theta_{v}^{⊤}\theta_{v}^{\prime }+\mathrm{tr}\left({\theta_{M}^{\prime }}^{⊤}\theta_{M}\right),$$

where tr(·) denotes the matrix trace, we rewrite the MVN density of Equation (83) in the canonical form of an exponential family [37]:

(85) $$\begin{array}{ccc} p_{\theta }\left(x;\theta \right) & := & \exp\left(\langle t(x),\theta \rangle -F_{\theta }\left(\theta \right)\right)=p_{\lambda }\left(x;\lambda \left(\theta \right)\right), \end{array}$$

where

(86) $$\theta =\left(\theta_{v},\theta_{M}\right)=\left(\Sigma^{-1}\mu ,-\frac{1}{2}\Sigma^{-1}\right)=\theta \left(\lambda \right)=\left(\lambda_{M}^{-1}\lambda_{v},-\frac{1}{2}\lambda_{M}^{-1}\right),$$

is the *compound natural parameter* and

(87) $$t\left(x\right)=\left(x,-xx^{⊤}\right)$$

is the *compound sufficient statistic*. The function $F_{\theta }$ is the strictly convex and continuously differentiable log-normalizer defined by:

(88) $$F_{\theta }\left(\theta \right)=\frac{1}{2}\left(d\log\pi -\log|\theta_{M}|+\frac{1}{2}\theta_{v}^{⊤}\theta_{M}^{-1}\theta_{v}\right),$$

The log-normalizer can be expressed using the ordinary parameters, λ=(μ,Σ), as:

(89) $$\begin{array}{ccc} F_{\lambda }\left(\lambda \right) & = & \frac{1}{2}\left(\lambda_{v}^{⊤}\lambda_{M}^{-1}\lambda_{v}+\log\left|\lambda_{M}\right|+d\log2\pi \right), \end{array}$$

(90) $$\begin{array}{cc} = & \frac{1}{2}\left(\mu^{⊤}\Sigma^{-1}\mu +\log\left|\Sigma \right|+d\log2\pi \right). \end{array}$$

The *moment/expectation parameters* [18,49] are

(91) $$\eta =\left(\eta_{v},\eta_{M}\right)=E\left[t\left(x\right)\right]=\nabla F\left(\theta \right).$$

We report the conversion formula between the three types of coordinate systems (namely the ordinary parameter λ, the natural parameter θ and the moment parameter η) as follows:

(92) $$\begin{array}{ccc} \left\{\begin{array}{c} \theta_{v}\left(\lambda \right)=\lambda_{M}^{-1}\lambda_{v}=\Sigma^{-1}\mu \\ \theta_{M}\left(\lambda \right)=\frac{1}{2}\lambda_{M}^{-1}=\frac{1}{2}\Sigma^{-1} \end{array}\right. & \Leftrightarrow & \left\{\begin{array}{c} \lambda_{v}\left(\theta \right)=\frac{1}{2}\theta_{M}^{-1}\theta_{v}=\mu \\ \lambda_{M}\left(\theta \right)=\frac{1}{2}\theta_{M}^{-1}=\Sigma \end{array}\right. \end{array}$$

(93) $$\begin{array}{ccc} \left\{\begin{array}{c} \eta_{v}\left(\theta \right)=\frac{1}{2}\theta_{M}^{-1}\theta_{v}\\ \eta_{M}\left(\theta \right)=-\frac{1}{2}\theta_{M}^{-1}-\frac{1}{4}\left(\theta_{M}^{-1}\theta_{v}\right){\left(\theta_{M}^{-1}\theta_{v}\right)}^{⊤} \end{array}\right. & \Leftrightarrow & \left\{\begin{array}{c} \theta_{v}\left(\eta \right)=-{\left(\eta_{M}+\eta_{v}\eta_{v}^{⊤}\right)}^{-1}\eta_{v}\\ \theta_{M}\left(\eta \right)=-\frac{1}{2}{\left(\eta_{M}+\eta_{v}\eta_{v}^{⊤}\right)}^{-1} \end{array}\right. \end{array}$$

(94) $$\begin{array}{ccc} \left\{\begin{array}{c} \lambda_{v}\left(\eta \right)=\eta_{v}=\mu \\ \lambda_{M}\left(\eta \right)=-\eta_{M}-\eta_{v}\eta_{v}^{⊤}=\Sigma \end{array}\right. & \Leftrightarrow & \left\{\begin{array}{c} \eta_{v}\left(\lambda \right)=\lambda_{v}=\mu \\ \eta_{M}\left(\lambda \right)=-\lambda_{M}-\lambda_{v}\lambda_{v}^{⊤}=-\Sigma -\mu \mu^{⊤} \end{array}\right. \end{array}$$

The dual Legendre convex conjugate [18,49] is

(95) $$F_{\eta }^{*}\left(\eta \right)=-\frac{1}{2}\left(\log\left(1+\eta_{v}^{⊤}\eta_{M}^{-1}\eta_{v}\right)+\log\left|-\eta_{M}\right|+d\left(1+\log2\pi \right)\right),$$

and $\theta =\nabla_{\eta }F_{\eta }^{*}\left(\eta \right)$.

We check the Fenchel-Young equality when η=∇F(θ) and $\theta =\nabla F^{*}\left(\eta \right)$:

(96) $$F_{\theta }\left(\theta \right)+F_{\eta }^{*}\left(\eta \right)-\langle \theta ,\eta \rangle =0.$$

The Kullback–Leibler divergence between two *d*-dimensional Gaussians distributions $p_{\left(\mu_{1},\Sigma_{1}\right)}$ and $p_{\left(\mu_{2},\Sigma_{2}\right)}$ (with $\Delta_{\mu }=\mu_{2}-\mu_{1}$) is

(97) $$\begin{array}{ccc} \mathrm{KL}(p_{\left(\mu_{1},\Sigma_{1}\right)}:p_{\left(\mu_{2},\Sigma_{2}\right)}) & = & \frac{1}{2}\left\{\mathrm{tr}\left(\Sigma_{2}^{-1}\Sigma_{1}\right)+\Delta_{\mu }^{⊤}\Sigma_{2}^{-1}\Delta_{\mu }+\log\frac{|\Sigma_{2}|}{|\Sigma_{1}|}-d\right\}=\mathrm{KL}\left(p_{\lambda_{1}}:p_{\lambda_{2}}\right). \end{array}$$

We check that $\mathrm{KL}(p_{\left(\mu ,\Sigma \right)}:p_{\left(\mu ,\Sigma \right)})=0$ since $\Delta_{\mu }=0$ and $\mathrm{tr}\left(\Sigma^{-1}\Sigma \right)=\mathrm{tr}\left(I\right)=d$. Notice that when $\Sigma_{1}=\Sigma_{2}=\Sigma$, we have

(98) $$\mathrm{KL}\left(p_{\left(\mu_{1},\Sigma \right)}:p_{\left(\mu_{2},\Sigma \right)}\right)=\frac{1}{2}\Delta_{\mu }^{⊤}\Sigma^{-1}\Delta_{\mu }=\frac{1}{2}D_{\Sigma^{-1}}^{2}\left(\mu_{1},\mu_{2}\right),$$

that is half the squared Mahalanobis distance for the precision matrix $\Sigma^{-1}$ (a positive-definite matrix: $\Sigma^{-1}≻0$), where the Mahalanobis distance is defined for any positive matrix Q≻0 as follows:

(99) $$D_{Q}\left(p_{1}:p_{2}\right)=\sqrt{{\left(p_{1}-p_{2}\right)}^{⊤}Q\left(p_{1}-p_{2}\right)}.$$

The Kullback–Leibler divergence between two probability densities of the same exponential families amount to a Bregman divergence [18]:

(100) $$\mathrm{KL}\left(p_{\left(\mu_{1},\Sigma_{1}\right)}:p_{\left(\mu_{2},\Sigma_{2}\right)}\right)=\mathrm{KL}\left(p_{\lambda_{1}}:p_{\lambda_{2}}\right)=B_{F}\left(\theta_{2}:\theta_{1}\right)=B_{F^{*}}\left(\eta_{1}:\eta_{2}\right),$$

where the Bregman divergence is defined by

(101) $$B_{F}\left(\theta :\theta^{\prime }\right):=F\left(\theta \right)-F\left(\theta^{\prime }\right)-\langle \theta -\theta^{\prime },\nabla F\left(\theta^{\prime }\right)\rangle ,$$

with $\eta^{\prime }=\nabla F\left(\theta^{\prime }\right)$. Define the canonical divergence [18]

(102) $$A_{F}\left(\theta_{1}:\eta_{2}\right)=F\left(\theta_{1}\right)+F^{*}\left(\eta_{2}\right)-\langle \theta_{1},\eta_{2}\rangle =A_{F^{*}}\left(\eta_{2}:\theta_{1}\right),$$

since ${F^{*}}^{*}=F$. We have $B_{F}\left(\theta_{1}:\theta_{2}\right)=A_{F}\left(\theta_{1}:\eta_{2}\right)$.

Now, observe that $p_{\theta }\left(0,\theta \right)=\exp\left(-F\left(\theta \right)\right)$ when 〈t(0),θ〉=0. In particular, this holds for the multivariate normal family. Thus, we have the following proposition.

**Proposition** **1.**  *For the MVN family, we have*

(103) $$p_{\theta }\left(x;{\left(\theta_{1}\theta_{2}\right)}_{\alpha }\right)=\frac{p_{\theta }{\left(x,\theta_{1}\right)}^{1-\alpha }p_{\theta }{\left(x,\theta_{2}\right)}^{\alpha }}{Z_{\alpha }^{G}\left(p_{\theta_{1}}:p_{\theta_{2}}\right)},$$

*with the scaling normalization factor:*

(104) $$Z_{\alpha }^{G}\left(p_{\theta_{1}}:p_{\theta_{2}}\right)=\exp\left(-J_{F}^{\alpha }\left(\theta_{1}:\theta_{2}\right)\right)=\frac{p_{\theta }{\left(0;\theta_{1}\right)}^{1-\alpha }p_{\theta }{\left(0;\theta_{2}\right)}^{\alpha }}{p_{\theta }\left(0;{\left(\theta_{1}\theta_{2}\right)}_{\alpha }\right)}.$$

More generally, we have for a *k*-dimensional weight vector α belonging to the (k−1)-dimensional standard simplex:

(105) $$Z_{\alpha }^{G}\left(p_{\theta_{1}},\ldots p_{\theta_{k}}\right)=\frac{\prod_{i=1}^{k}p_{\theta }{\left(0,\theta_{i}\right)}^{\alpha_{i}}}{p_{\theta }\left(0;\bar{\theta }\right)},$$

where $\bar{\theta }=\sum_{i=1}^{k}\alpha_{i}\theta_{i}$.

Finally, we state the formulas for the G-JS divergence between MVNs for the KL and reverse KL, respectively:

**Corollary** **1** (*G*-JSD between Gaussians)**.** The skew G-Jensen–Shannon divergence ${\mathrm{JS}}_{\alpha }^{G}$ and the dual skew G-Jensen–Shannon divergence ${{\mathrm{JS}}^{*}}_{\alpha }^{G}$ between two multivariate Gaussians $N(\mu_{1},\Sigma_{1})$ and $N(\mu_{2},\Sigma_{2})$ is

(106) $$\begin{array}{ccc} {\mathrm{JS}}^{G_{\alpha }}\left(p_{\left(\mu_{1},\Sigma_{1}\right)}:p_{\left(\mu_{2},\Sigma_{2}\right)}\right) & = & \left(1-\alpha \right)\mathrm{KL}\left(p_{\left(\mu_{1},\Sigma_{1}\right)}:p_{\left(\mu_{\alpha },\Sigma_{\alpha }\right)}\right)+\alpha \mathrm{KL}\left(p_{\left(\mu_{2},\Sigma_{2}\right)}:p_{\left(\mu_{\alpha },\Sigma_{\alpha }\right)}\right), \end{array}$$

(107) $$\begin{array}{ccc} & = & \left(1-\alpha \right)B_{F}\left({\left(\theta_{1}\theta_{2}\right)}_{\alpha }:\theta_{1}\right)+\alpha B_{F}\left({\left(\theta_{1}\theta_{2}\right)}_{\alpha }:\theta_{2}\right),\\ & = & \frac{1}{2}\left(\mathrm{tr}\left(\Sigma_{\alpha }^{-1}\left(\left(1-\alpha \right)\Sigma_{1}+\alpha \Sigma_{2}\right)\right)+\log\frac{|\Sigma_{\alpha }|}{|\Sigma_{1}{|}^{1-\alpha }{\left|\Sigma_{2}\right|}^{\alpha }}+\right. \end{array}$$

(108) $$\begin{array}{ccc} & & \left.\left(1-\alpha \right){\left(\mu_{\alpha }-\mu_{1}\right)}^{⊤}\Sigma_{\alpha }^{-1}\left(\mu_{\alpha }-\mu_{1}\right)+\alpha {\left(\mu_{\alpha }-\mu_{2}\right)}^{⊤}\Sigma_{\alpha }^{-1}\left(\mu_{\alpha }-\mu_{2}\right)-d\right) \end{array}$$

(109) $$\begin{array}{ccc} {\mathrm{JS}}_{*}^{G_{\alpha }}\left(p_{\left(\mu_{1},\Sigma_{1}\right)}:p_{\left(\mu_{2},\Sigma_{2}\right)}\right) & = & \left(1-\alpha \right)\mathrm{KL}\left(p_{\left(\mu_{\alpha },\Sigma_{\alpha }\right)}:p_{\left(\mu_{1},\Sigma_{1}\right)}\right)+\alpha \mathrm{KL}\left(p_{\left(\mu_{\alpha },\Sigma_{\alpha }\right)}:p_{\left(\mu_{2},\Sigma_{2}\right)}\right), \end{array}$$

(110) $$\begin{array}{ccc} & = & \left(1-\alpha \right)B_{F}\left(\theta_{1}:{\left(\theta_{1}\theta_{2}\right)}_{\alpha }\right)+\alpha B_{F}\left(\theta_{2}:{\left(\theta_{1}\theta_{2}\right)}_{\alpha }\right), \end{array}$$

(111) $$\begin{array}{ccc} & = & J_{F}\left(\theta_{1}:\theta_{2}\right), \end{array}$$

(112) $$\begin{array}{ccc} & = & \frac{1}{2}\left(\left(1-\alpha \right)\mu_{1}^{⊤}\Sigma_{1}^{-1}\mu_{1}+\alpha \mu_{2}^{⊤}\Sigma_{2}^{-1}\mu_{2}-\mu_{\alpha }^{⊤}\Sigma_{\alpha }^{-1}\mu_{\alpha }+\log\frac{|\Sigma_{1}{|}^{1-\alpha }{\left|\Sigma_{2}\right|}^{\alpha }}{|\Sigma_{\alpha }|}\right), \end{array}$$

*where*

(113) $$\Sigma_{\alpha }={\left(\Sigma_{1}\Sigma_{2}\right)}_{\alpha }^{\Sigma }={\left(\left(1-\alpha \right)\Sigma_{1}^{-1}+\alpha \Sigma_{2}^{-1}\right)}^{-1},$$

*(matrix harmonic barycenter) and*

(114) $$\mu_{\alpha }={\left(\mu_{1}\mu_{2}\right)}_{\alpha }^{\mu }=\Sigma_{\alpha }\left(\left(1-\alpha \right)\Sigma_{1}^{-1}\mu_{1}+\alpha \Sigma_{2}^{-1}\mu_{2}\right).$$

Notice that the *α-skew Bhattacharyya distance* [7]:

(115) $$B_{\alpha }\left(p:q\right)=-\log\int_{X}p^{1-\alpha }q^{\alpha }d\mu$$

between two members of the same exponential family amounts to a α-skew Jensen divergence between the corresponding natural parameters:

(116) $$B_{\alpha }\left(p_{\theta_{1}}:p_{\theta_{2}}\right)=J_{F}^{\alpha }\left(\theta_{1}:\theta_{2}\right).$$

A simple proof follows from the fact that

(117) $$\int p_{{\left(\theta_{1}\theta_{2}\right)}_{\alpha }}\left(x\right)d\mu \left(x\right)=1=\int \frac{p_{\theta_{1}}^{1-\alpha }\left(x\right)p_{\theta_{2}}^{\alpha }\left(x\right)}{Z_{\alpha }^{G}\left(p_{\theta_{1}}:p_{\theta_{2}}\right)}d\mu \left(x\right).$$

Therefore, we have

(118) $$\log1=0=\log\int p_{\theta_{1}}^{1-\alpha }\left(x\right)p_{\theta_{2}}^{\alpha }\left(x\right)d\mu \left(x\right)-\log Z_{\alpha }^{G}\left(p_{\theta_{1}}:p_{\theta_{2}}\right),$$

with $Z_{\alpha }^{G}\left(p_{\theta_{1}}:p_{\theta_{2}}\right)=\exp\left(-J_{F}\left(p_{\theta_{1}}:p_{\theta_{2}}\right)\right)$. Thus, it follows that

(119) $$\begin{array}{ccc} B_{\alpha }\left(p_{\theta_{1}}:p_{\theta_{2}}\right) & = & -\log\int p_{\theta_{1}}^{1-\alpha }\left(x\right)p_{\theta_{2}}^{\alpha }\left(x\right)d\mu \left(x\right), \end{array}$$

(120) $$\begin{array}{cc} = & -\log Z_{\alpha }^{G}\left(p_{\theta_{1}}:p_{\theta_{2}}\right), \end{array}$$

(121) $$\begin{array}{cc} = & J_{F}\left(p_{\theta_{1}}:p_{\theta_{2}}\right). \end{array}$$

**Corollary** **2.**  *The JS-symmetrization of the reverse Kullback–Leibler divergence between densities of the same exponential family amount to calculate a Jensen/Burbea–Rao divergence between the corresponding natural parameters.*

#### 4.1.2. Applications to *k*-Means Clustering

Let $P=\{p_{1},\ldots ,p_{n}\}$ denote a point set, and $C=\{c_{1},\ldots ,c_{k}\}$ denote a set of *k* (cluster) centers. The generalized *k*-means objective [23] with respect to a distance *D* is defined by:

(122) $$E_{D}\left(P,C\right)=\frac{1}{n}\sum_{i=1}^{n}\min_{j\in \{1,\ldots ,k\}}D\left(p_{i}:c_{j}\right).$$

By defining the distance $D\left(p,C\right)=\min_{j\in \{1,\ldots ,k\}}D\left(p:c_{j}\right)$ of a point to a set of points, we can rewrite compactly the objective function as $E_{D}\left(P,C\right)=\frac{1}{n}\sum_{i=1}^{n}D\left(p_{i},C\right)$. Denote by $E_{D}^{*}\left(P,k\right)$ the minimum objective loss for a set of k=|C| clusters: $E_{D}^{*}\left(P,k\right)=\min_{|C|=k}E_{D}\left(P,C\right)$. It is NP-hard [50] to compute $E_{D}^{*}\left(P,k\right)$ when k>1 and the dimension d>1. The most common heuristic is Lloyd’s batched *k*-means [23] that yields a local minimum.

The performance of the *probabilistic k-means++ initialization* [51] has been extended to arbitrary distances in [52] as follows:

**Theorem** **3**  (Generalized k-means++ performance, [53])**.** *Let $\kappa_{1}$ and $\kappa_{2}$ be two constants such that $\kappa_{1}$ defines the quasi-triangular inequality property:*

(123) $$D\left(x:z\right)\leq \kappa_{1}\left(D(x:y)+D(y:z)\right),\;\forall x,y,z\in \Delta^{d},$$

*and $\kappa_{2}$ handles the symmetry inequality:*

(124) $$D\left(x:y\right)\leq \kappa_{2}D\left(y:x\right),\;\forall x,y\in \Delta^{d}.$$

*Then the generalized k-means++ seeding guarantees with high probability a configuration C of cluster centers such that:*

(125) $$E_{D}\left(P,C\right)\leq 2\kappa_{1}^{2}\left(1+\kappa_{2}\right)\left(2+\log k\right)E_{D}^{*}\left(P,k\right).$$

To bound the constants $\kappa_{1}$ and $\kappa_{2}$, we rewrite the generalized Jensen–Shannon divergences using quadratic form expressions: That is, using a squared Mahalanobis distance:

(126) $$D_{Q}\left(p:q\right)=\sqrt{{\left(p-q\right)}^{⊤}Q\left(p-q\right)},$$

for a positive-definite matrix Q≻0. Since the Bregman divergence can be interpreted as the tail of a first-order Taylor expansion, we have:

(127) $$B_{F}\left(\theta_{1}:\theta_{2}\right)=\frac{1}{2}{\left(\theta_{1}-\theta_{2}\right)}^{⊤}\nabla^{2}F\left(\xi \right)\left(\theta_{1}-\theta_{2}\right),$$

for ξ∈Θ (open convex). Similarly, the Jensen divergence can be interpreted as a Jensen–Bregman divergence, and thus we have

(128) $$J_{F}\left(\theta_{1}:\theta_{2}\right)\frac{1}{2}{\left(\theta_{1}-\theta_{2}\right)}^{⊤}\nabla^{2}F\left(\xi^{\prime }\right)\left(\theta_{1}-\theta_{2}\right),$$

for $\xi^{\prime }\in \Theta$. More precisely, for a prescribed point set $\left\{\theta_{1},\ldots ,\theta_{n}\right\}$, we have $\xi ,\xi^{\prime }\in \mathrm{CH}\left(\left\{\theta_{1},\ldots ,\theta_{n}\right\}\right)$, where CH denotes the closed convex hull. We can therefore upper bound $\kappa_{1}$ and $\kappa_{2}$ using the ratio $\frac{\max_{\theta \in \mathrm{CH}(\left\{\theta_{1},\ldots ,\theta_{n}\right\})}∥\nabla^{2}F\left(\theta \right)∥}{\max_{\theta \in \mathrm{CH}(\left\{\theta_{1},\ldots ,\theta_{n}\right\})}∥\nabla^{2}F\left(\theta \right)∥}$. See [54] for further details.

A centroid for a set of parameters $\theta_{1},\ldots ,\theta_{n}$ is defined as the minimizer of the functional

(129) $$E_{D}\left(\theta \right)=\frac{1}{n}\sum_{i}D\left(\theta_{i}:\theta \right).$$

In particular, the *symmetrized Bregman centroids* have been studied in [55] (for ${\mathrm{JS}}^{G_{\alpha }}$), and the *Jensen centroids* (for ${\mathrm{JS}}_{*}^{G_{\alpha }}$) have been investigated in [7] using the convex-concave iterative procedure.

### 4.2. The Harmonic Jensen–Shannon Divergence (H-JS)

The *principle* to get closed-form formula for generalized Jensen–Shannon divergences between distributions belonging to a parametric family $P_{\Theta }=\left\{p_{\theta }\;:\;\theta \in \Theta \right\}$ consists of finding an abstract mean *M* such that the *M*-mixture ${\left(p_{\theta_{1}}p_{\theta_{2}}\right)}_{\alpha }^{M}$ belongs to the family $P_{\Theta }$. In particular, when Θ is a convex domain, we seek a mean *M* such that ${\left(p_{\theta_{1}}p_{\theta_{2}}\right)}_{\alpha }^{M}=p_{{\left(\theta_{1}\theta_{2}\right)}_{\alpha }}$ with ${\left(\theta_{1}\theta_{2}\right)}_{\alpha }\in \Theta$.

Let us consider the *weighted harmonic mean* [34] (induced by the harmonic mean) *H*:

(130) $$H_{\alpha }\left(x,y\right):=\frac{1}{\left(1-\alpha \right)\frac{1}{x}+\alpha \frac{1}{y}}=\frac{xy}{(1-\alpha )y+\alpha x}=\frac{xy}{{\left(xy\right)}_{1-\alpha }},\;\alpha \in \left[0,1\right].$$

The harmonic mean is a quasi-arithmetic mean $H_{\alpha }\left(x,y\right)=M_{\alpha }^{h}\left(x,y\right)$ obtained for the monotone (decreasing) function $h\left(u\right)=\frac{1}{u}$ (or equivalently for the increasing monotone function $h\left(u\right)=-\frac{1}{u}$).

This harmonic mean is well-suited for the *scale family* C of Cauchy probability distributions (also called Lorentzian distributions):

(131) $$C_{\Gamma }:=\left\{\;p_{\gamma }\left(x\right)=\frac{1}{\gamma }p_{\mathrm{std}}\left(\frac{x}{\gamma }\right)=\frac{\gamma }{\pi (\gamma^{2}+x^{2})}\;:\;\gamma \in \Gamma =\left(0,\infty \right)\right\},$$

where γ denotes the scale and $p_{\mathrm{std}}\left(x\right)=\frac{1}{\pi (1+x^{2})}$ the *standard Cauchy distribution*.

Using the computer algebra system Maxima (http://maxima.sourceforge.net/) we find that (see Appendix B)

(132) $${\left(p_{\gamma_{1}}p_{\gamma_{2}}\right)}_{\frac{1}{2}}^{H}\left(x\right)=\frac{H_{\alpha }\left(p_{\gamma_{1}}\left(x\right):p_{\gamma_{2}}\left(x\right)\right)}{Z_{\alpha }^{H}\left(\gamma_{1},\gamma_{2}\right)}=p_{{\left(\gamma_{1}\gamma_{2}\right)}_{\alpha }}$$

where the normalizing coefficient is

(133) $$Z_{\alpha }^{H}\left(\gamma_{1},\gamma_{2}\right):=\sqrt{\frac{\gamma_{1}\gamma_{2}}{{\left(\gamma_{1}\gamma_{2}\right)}_{\alpha }{\left(\gamma_{1}\gamma_{2}\right)}_{1-\alpha }}}=\sqrt{\frac{\gamma_{1}\gamma_{2}}{{\left(\gamma_{1}\gamma_{2}\right)}_{\alpha }{\left(\gamma_{2}\gamma_{1}\right)}_{\alpha }}},$$

since we have ${\left(\gamma_{1}\gamma_{2}\right)}_{1-\alpha }={\left(\gamma_{2}\gamma_{1}\right)}_{\alpha }$.

The *H*-Jensen–Shannon symmetrization of a distance *D* between distributions writes as:

(134) $${\mathrm{JS}}_{D}^{H_{\alpha }}\left(p:q\right)=\left(1-\alpha \right)D\left(p:{\left(pq\right)}_{\alpha }^{H}\right)+\alpha D\left(q:{\left(pq\right)}_{\alpha }^{H}\right),$$

where $H_{\alpha }$ denote the weighted harmonic mean. When *D* is available in closed form for distributions belonging to the scale Cauchy distributions, so is ${\mathrm{JS}}_{D}^{H_{\alpha }}\left(p:q\right)$.

For example, consider the KL divergence formula between two scale Cauchy distributions:

(135) $$\mathrm{KL}\left(p_{\gamma_{1}}:p_{\gamma_{2}}\right)=2\log\frac{A(\gamma_{1},\gamma_{2})}{G(\gamma_{1},\gamma_{2})}=2\log\frac{\gamma_{1}+\gamma_{2}}{2\sqrt{\gamma_{1}\gamma_{2}}},$$

where *A* and *G* denote the arithmetic and geometric means, respectively. The formula initially reported in [56] has been corrected by the authors. Since A≥G (and $\frac{A}{G}\geq 1$), it follows that $\mathrm{KL}(p_{\gamma_{1}}:p_{\gamma_{2}})\geq 0$. Notice that the KL divergence is symmetric for Cauchy scale distributions. We note in passing that for exponential families, the KL divergence is symmetric only for the location Gaussian family (since the only symmetric Bregman divergences are the squared Mahalanobis distances [57]). The cross-entropy between scale Cauchy distributions is $h^{\times }\left(p_{\gamma_{1}}:p_{\gamma_{2}}\right)=\log\pi \frac{{\left(\gamma_{1}+\gamma_{2}\right)}^{2}}{\gamma_{2}}$, and the differential entropy is $h\left(p_{\gamma }\right)=h^{\times }\left(p_{\gamma }:p_{\gamma }\right)=\log4\pi \gamma$.

Then the *H*-JS divergence between $p=p_{\gamma_{1}}$ and $q=p_{\gamma_{2}}$ is:

(136) $$\begin{array}{ccc} {\mathrm{JS}}^{H}\left(p:q\right) & = & \frac{1}{2}\left(\mathrm{KL}\left(p:{\left(pq\right)}_{\frac{1}{2}}^{H}\right)+\mathrm{KL}\left(q:{\left(pq\right)}_{\frac{1}{2}}^{H}\right)\right), \end{array}$$

(137) $$\begin{array}{ccc} {\mathrm{JS}}^{H}\left(p_{\gamma_{1}}:p_{\gamma_{2}}\right) & = & \frac{1}{2}\left(\mathrm{KL}\left(p_{\gamma_{1}}:p_{\frac{\gamma_{1}+\gamma_{2}}{2}}\right)+\mathrm{KL}\left(p_{\gamma_{2}}:p_{\frac{\gamma_{1}+\gamma_{2}}{2}}\right)\right), \end{array}$$

(138) $$\begin{array}{cc} = & \log\frac{\left(3\gamma_{1}+\gamma_{2}\right)\left(3\gamma_{2}+\gamma_{1}\right)}{8\sqrt{\gamma_{1}\gamma_{2}}\left(\gamma_{1}+\gamma_{2}\right)}. \end{array}$$

We check that when $\gamma_{1}=\gamma_{2}=\gamma$, we have ${\mathrm{JS}}^{H_{\alpha }}\left(p_{\gamma }:p_{\gamma }\right)=0$.

**Theorem** **4** (Harmonic JSD between scale Cauchy distributions)**.** *The harmonic Jensen–Shannon divergence between two scale Cauchy distributions $p_{\gamma_{1}}$ and $p_{\gamma_{2}}$ is ${\mathrm{JS}}^{H}\left(p_{\gamma_{1}}:p_{\gamma_{2}}\right)=\log\frac{\left(3\gamma_{1}+\gamma_{2}\right)\left(3\gamma_{2}+\gamma_{1}\right)}{8\sqrt{\gamma_{1}\gamma_{2}}\left(\gamma_{1}+\gamma_{2}\right)}$.*

Let us report some numerical examples: Consider $p_{\gamma_{1}}=0.1$ and $p_{\gamma_{1}}=0.5$, we find that ${\mathrm{JS}}^{H}\left(p_{\gamma_{1}}\;:\;p_{\gamma_{2}}\right)≃0.176$. When $p_{\gamma_{1}}=0.2$ and $p_{\gamma_{1}}=0.8$, we find that ${\mathrm{JS}}^{H}\left(p_{\gamma_{1}}:p_{\gamma_{2}}\right)≃0.129$.

Notice that KL formula is scale-invariant, and this property holds for any scale family:

**Lemma** **1.**  *The Kullback–Leibler divergence between two distributions $p_{s_{1}}$ and $p_{s_{2}}$ belonging to the same scale family ${\left\{p_{s}\left(x\right)=\frac{1}{s}p\left(\frac{x}{s}\right)\right\}}_{s\in (0,\infty )}$ with standard density p is scale-invariant: $\mathrm{KL}\left(p_{\lambda s_{1}}:\;p_{\lambda s_{2}}\right)\;=\;\mathrm{KL}\left(p_{s_{1}}:\;p_{s_{2}}\right)=\mathrm{KL}\left(p:p_{\frac{s_{2}}{s_{1}}}\right)=\mathrm{KL}\left(p_{\frac{s_{1}}{s_{2}}}:p\right)$ for any λ>0.*

A direct proof follows from a change of variable in the KL integral with $y=\frac{x}{\lambda }$ and dx=λdy. Please note that although the KLD between scale Cauchy distributions is symmetric, it is not the case for all scale families: For example, the Rayleigh distributions form a scale family with the KLD amounting to compute a Bregman asymmetric Itakura–Saito divergence between parameters [37].

Instead of the KLD, we can choose the total variation distance for which a formula has been reported in [38] between two Cauchy distributions. Notice that the Cauchy distributions are alpha-stable distributions for α=1 and *q* Gaussian distributions for q=2 ([58], p. 104). A closed-form formula for the divergence between two *q*-Gaussians is given in [58] when q<2. The definite integral $h_{q}\left(p\right)=\int_{-\infty }^{+\infty }p{\left(x\right)}^{q}d\mu$ is available in closed form for Cauchy distributions. When q=2, we have $h_{2}\left(p_{\gamma }\right)=\frac{1}{2\pi \gamma }$.

We refer to [38] for yet other illustrative examples considering the family of Pearson type VII distributions and central multivariate *t*-distributions which use the power means (quasi-arithmetic means $M^{h}$ induced by $h\left(u\right)=u^{\alpha }$ for α>0) for defining mixtures.

Table 1 summarizes the various examples introduced in the paper.

### 4.3. The M-Jensen–Shannon Matrix Distances

In this section, we consider distances between matrices which play an important role in quantum computing [59,60]. We refer to [61] for the matrix Jensen–Bregman logdet divergence. The *Hellinger distance* can be interpreted as the difference of an arithmetic mean *A* and a geometric mean *G*:

(139) $$D_{H}\left(p,q\right)=\sqrt{1-\int_{X}\sqrt{p(x)}\sqrt{q(x)}d\mu \left(x\right)}=\sqrt{\int_{X}\left(A\left(p\left(x\right),q\left(x\right)\right)-G\left(p\left(x\right),q\left(x\right)\right)\right)d\mu \left(x\right)}.$$

Notice that since A≥G, we have $D_{H}\left(p,q\right)\geq 0$. The scaled and squared Hellinger distance is an α-divergence $I_{\alpha }$ for α=0. Recall that the α-divergence can be interpreted as the difference of a weighted arithmetic minus a weighted geometry mean.

In general, if a mean $M_{1}$ dominates a mean $M_{2}$, we may define the distance as

(140) $$D_{M_{1},M_{2}}\left(p,q\right)=\int_{X}\left(M_{1}\left(p,q\right)-M_{2}\left(p,q\right)\right)d\mu \left(x\right).$$

When considering matrices [62], there is *not* a unique definition of a geometric matrix mean, and thus we have different notions of matrix Hellinger distances [62], some of them are divergences (smooth distances defining a dualistic structure in information geometry).

We define the *matrix M-Jensen–Shannon divergence* for a matrix divergence [63,64] *D* as follows:

(141) $${\mathrm{JS}}_{D}^{M}\left(X_{1},X_{2}\right)=\frac{1}{2}\left(D\left(X_{1}:M\left(X_{1},X_{2}\right)\right)+D\left(X_{2}:M\left(X_{1},X_{2}\right)\right)\right)={\mathrm{JS}}_{D}^{M}\left(X_{2},X_{1}\right).$$

For example, we can choose the *von Neumann matrix divergence* [63]:

(142) $$D_{\mathrm{vN}}\left(X_{1}:X_{2}\right):=\mathrm{tr}\left(X_{1}\log X_{1}-X_{1}\log X_{2}-X_{1}+X_{2}\right),$$

or the *LogDet matrix divergence* [63]:

(143) $$D_{\mathrm{ld}}\left(X_{1}:X_{2}\right):=\mathrm{tr}\left(X_{1}X_{2}^{-1}\right)-\log\left|X_{1}X_{2}^{-1}\right|-d,$$

where square matrices $X_{1}$ and $X_{2}$ have dimension *d*.

## 5. Conclusions and Perspectives

We introduced a generalization of the celebrated Jensen–Shannon divergence [6], termed the *(M,N)-Jensen–Shannon divergences*, based on *M-mixtures* derived from abstract means *M*. This new family of divergences includes the ordinary Jensen–Shannon divergence when both *M* and *N* are set to the arithmetic mean. We reported closed-form expressions of the *M* Jensen–Shannon divergences for mixture families and exponential families in information geometry by choosing the arithmetic and geometric weighted mean, respectively. The α-skewed geometric Jensen–Shannon divergence (*G*-Jensen–Shannon divergence) between densities $p_{\theta_{1}}$ and $p_{\theta_{2}}$ of the same exponential family with cumulant function *F* is

$${\mathrm{JS}}_{\mathrm{KL}}^{G_{\alpha }}\left[p_{\theta_{1}}:p_{\theta_{2}}\right]={\mathrm{JS}}_{{B_{F}}^{*}}^{A_{\alpha }}\left(\theta_{1}:\theta_{2}\right).$$

Here, we used the bracket notation to emphasize that the statistical distance ${\mathrm{JS}}_{\mathrm{KL}}^{G_{\alpha }}$ is between densities, and the parenthesis notation to emphasize that the distance ${\mathrm{JS}}_{{B_{F}}^{*}}^{A_{\alpha }}$ is between parameters. We also have ${\mathrm{JS}}_{{\mathrm{KL}}^{*}}^{G_{\alpha }}\left[p_{\theta_{1}}:p_{\theta_{2}}\right]=J_{F}^{\alpha }\left(\theta_{1}:\theta_{2}\right)$. We also show how to get a closed-form formula for the harmonic Jensen–Shannon divergence of Cauchy scale distributions by taking harmonic mixtures.

For an arbitrary distance *D*, we define the *skew N-Jeffreys symmetrization*:

(144) $$J_{D}^{N_{\beta }}\left(p_{1}:p_{2}\right)=N_{\beta }\left(D\left(p_{1}:p_{2}\right),D\left(p_{{}_{2}}:p_{1}\right)\right),$$

and the *skew (M,N)-JS-symmetrization*:

(145) $${\mathrm{JS}}_{D}^{M_{\alpha },N_{\beta }}\left(p_{1}:p_{2}\right)=N_{\beta }\left(D\left(p_{1}:{\left(p_{1}p_{2}\right)}_{\alpha }^{M}\right),D\left(p_{2}:{\left(p_{1}p_{2}\right)}_{\alpha }^{M}\right)\right).$$

A Java™ source code for computing the geometric Jensen–Shannon divergence between multivariate Gaussian distributions is available online at https://franknielsen.github.io/M-JS/.

## Figures and Tables

**Table 1:** Summary of the weighted means *M* chosen according to the parametric family in order to ensure that the family is closed under *M*-mixturing: ${\left(p_{\theta_{1}}p_{\theta_{2}}\right)}_{\alpha }^{M}=p_{{\left(\theta_{1}\theta_{2}\right)}_{\alpha }}$.

${\mathrm{JS}}^{M_{\alpha }}$	Mean *M*	Parametric Family	$Z_{\alpha }^{M}\left(p:q\right)$
${\mathrm{JS}}^{A_{\alpha }}$	arithmetic *A*	mixture family	$Z_{\alpha }^{M}\left(\theta_{1}:\theta_{2}\right)=1$
${\mathrm{JS}}^{G_{\alpha }}$	geometric *G*	exponential family	$Z_{\alpha }^{G}\left(\theta_{1}:\theta_{2}\right)=\exp\left(-J_{F}^{\alpha }\left(\theta_{1}:\theta_{2}\right)\right)$
${\mathrm{JS}}^{H_{\alpha }}$	harmonic *H*	Cauchy scale family	$Z_{\alpha }^{H}\left(\theta_{1}:\theta_{2}\right)=\sqrt{\frac{\theta_{1}\theta_{2}}{{\left(\theta_{1}\theta_{2}\right)}_{\alpha }{\left(\theta_{1}\theta_{2}\right)}_{1-\alpha }}}$

## Appendix A. Summary of Distances and Their Notations

Table A1 lists the main distances with their notations.

**Table A1:** Summary of Distances and Their Notations.

Weighted mean	$M_{\alpha }$, α∈(0,1)
Arithmetic mean	$A_{\alpha }\left(x,y\right)=\left(1-\alpha \right)x+\alpha y$
Geometric mean	$G_{\alpha }\left(x,y\right)=x^{1-\alpha }y^{\alpha }$
Harmonic mean	$H_{\alpha }\left(x,y\right)=\frac{xy}{(1-\alpha )y+\alpha x}$
Power mean	$P_{\alpha }^{p}\left(x,y\right)={\left(\left(1-\alpha \right)x^{p}+\alpha y^{p}\right)}^{\frac{1}{p}},\;p\in R\setminus \left\{0\right\}$, $\lim_{p\to 0}P_{\alpha }^{p}=G$
Quasi-arithmetic mean	$M_{\alpha }^{f}\left(x,y\right)=f^{-1}\left(\left(1-\alpha \right)f\left(x\right)+\alpha f\left(y\right)\right)$, *f* strictly monotonous
*M*-mixture	$Z_{\alpha }^{M}\left(p,q\right)=\int_{t\in X}M_{\alpha }\left(p\left(t\right),q\left(t\right)\right)d\mu \left(t\right)$ with $Z_{\alpha }^{M}\left(p,q\right)=\int_{t\in X}M_{\alpha }\left(p\left(t\right),q\left(t\right)\right)d\mu \left(t\right)$
**Statistical distance**	D(p:q)
Dual/reverse distance $D^{*}$	$D^{*}\left(p:q\right):=D\left(q:p\right)$
Kullback-Leibler divergence	$\mathrm{KL}\left(p:q\right)=\int p\left(x\right)\log\frac{p(x)}{q(x)}d\mu \left(x\right)$
reverse Kullback-Leibler divergence	${\mathrm{KL}}^{*}\left(p:q\right)=\mathrm{KL}\left(q:p\right)=\int q\left(x\right)\log\frac{q(x)}{p(x)}d\mu \left(x\right)$
Jeffreys divergence	$J\left(p;q\right)=\mathrm{KL}\left(p:q\right)+\mathrm{KL}\left(q:p\right)=\int \left(p\left(x\right)-q\left(x\right)\right)\log\frac{p(x)}{q(x)}d\mu \left(x\right)$
Resistor divergence	$\frac{1}{R(p;q)}=\frac{1}{2}\left(\frac{1}{\mathrm{KL}(p:q)}+\frac{1}{\mathrm{KL}(q:p)}\right)$. $R\left(p;q\right)=\frac{2J(p;q)}{\mathrm{KL}(p:q)\mathrm{KL}(q:p)}$
skew *K*-divergence	$K_{\alpha }\left(p:q\right)=\int p\left(x\right)\log\frac{p(x)}{\left(1-\alpha )p(x)+\alpha q(x\right)}d\mu \left(x\right)$
Jensen-Shannon divergence	$\mathrm{JS}\left(p,q\right)=\frac{1}{2}\left(\mathrm{KL}\left(p:\frac{p+q}{2}\right)+\mathrm{KL}\left(q:\frac{p+q}{2}\right)\right)$
skew Bhattacharrya divergence	$B_{\alpha }\left(p:q\right)=-\log\int_{X}p{\left(x\right)}^{1-\alpha }q{\left(x\right)}^{\alpha }d\mu \left(x\right)$
Hellinger distance	$D_{H}\left(p,q\right)=\sqrt{1-\int_{X}\sqrt{p(x)}\sqrt{q(x)}d\mu \left(x\right)}$
α-divergences	$I_{\alpha }\left(p:q\right)=\int \left(\alpha p\left(x\right)+\left(1-\alpha \right)q\left(x\right)-p{\left(x\right)}^{\alpha }q{\left(x\right)}^{1-\alpha }\right)d\mu \left(x\right),\alpha \notin \left\{0,1\right\}$ $I_{\alpha }\left(p:q\right)=A_{\alpha }\left(q:p\right)-G_{\alpha }\left(q:p\right)$
Mahalanobis distance	$D_{Q}\left(p:q\right)=\sqrt{{\left(p-q\right)}^{⊤}Q\left(p-q\right)}$ for a positive-definite matrix Q≻0
*f*-divergence	$I_{f}\left(p:q\right)=\int p\left(x\right)f\left(\frac{q(x)}{p(x)}\right)d\mu \left(x\right)$, with $f\left(1\right)=f^{\prime }\left(1\right)=0$ *f* strictly convex at 1
reverse *f*-divergence	$I_{f}^{*}\left(p:q\right)=\int q\left(x\right)f\left(\frac{p(x)}{q(x)}\right)d\mu \left(x\right)=I_{f^{⋄}}\left(p:q\right)$ for $f^{⋄}\left(u\right)=uf\left(\frac{1}{u}\right)$
J-symmetrized *f*-divergence	$J_{f}\left(p;q\right)=\frac{1}{2}\left(I_{f}\left(p:q\right)+I_{f}\left(q:p\right)\right)$
JS-symmetrized *f*-divergence	$I_{f}^{\alpha }\left(p;q\right):=\left(1-\alpha \right)I_{f}\left(p:{\left(pq\right)}_{\alpha }\right)+\alpha I_{f}\left(q:{\left(pq\right)}_{\alpha }\right)=I_{f_{\alpha }^{\mathrm{JS}}}\left(p:q\right)$ for $f_{\alpha }^{\mathrm{JS}}\left(u\right):=\left(1-\alpha \right)f\left(\alpha u+1-\alpha \right)+\alpha f\left(\alpha +\frac{1-\alpha }{u}\right)$
**Parameter distance**	
Bregman divergence	$B_{F}\left(\theta :\theta^{\prime }\right):=F\left(\theta \right)-F\left(\theta^{\prime }\right)-\langle \theta -\theta^{\prime },\nabla F\left(\theta^{\prime }\right)\rangle$
skew Jeffreys-Bregman divergence	$S_{F}^{\alpha }=\left(1-\alpha \right)B_{F}\left(\theta :\theta^{\prime }\right)+\alpha B_{F}\left(\theta^{\prime }:\theta \right)$
skew Jensen divergence	$J_{F}^{\alpha }\left(\theta :\theta^{\prime }\right):={\left(F\left(\theta \right)F\left(\theta^{\prime }\right)\right)}_{\alpha }-F\left({\left(\theta \theta^{\prime }\right)}_{\alpha }\right)$
Jensen-Bregman divergence	${\mathrm{JB}}_{F}\left(\theta ;\theta^{\prime }\right)=\frac{1}{2}\left(B_{F}\left(\theta :\frac{\theta +\theta^{\prime }}{2}\right)+B_{F}\left(\theta^{\prime }:\frac{\theta +\theta^{\prime }}{2}\right)\right)=J_{F}\left(\theta ;\theta^{\prime }\right)$.
**Generalized Jensen-Shannon divergences**	
skew *J*-symmetrization	$J_{D}^{\alpha }\left(p:q\right):=\left(1-\alpha \right)D\left(p:q\right)+\alpha D\left(q:p\right)$
skew JS-symmetrization	${\mathrm{JS}}_{D}^{\alpha }\left(p:q\right):=\left(1-\alpha \right)D\left(p:(1-\alpha )p+\alpha q\right)+\alpha D\left(q:(1-\alpha )p+\alpha q\right)$
skew *M*-Jensen-Shannon divergence	${\mathrm{JS}}^{M_{\alpha }}\left(p:q\right):=\left(1-\alpha \right)\mathrm{KL}\left(p:{\left(pq\right)}_{\alpha }^{M}\right)+\alpha \mathrm{KL}\left(q:{\left(pq\right)}_{\alpha }^{M}\right)$
skew *M*-JS-symmetrization	${\mathrm{JS}}_{D}^{M_{\alpha }}\left(p:q\right):=\left(1-\alpha \right)D\left(p:{\left(pq\right)}_{\alpha }^{M}\right)+\alpha D\left(q:{\left(pq\right)}_{\alpha }^{M}\right)$
*N*-Jeffreys divergence	$J^{N_{\beta }}\left(p:q\right):=N_{\beta }\left(\mathrm{KL}\left(p:q\right),\mathrm{KL}\left(q:p\right)\right)$
*N*-J D divergence	$J_{D}^{N_{\beta }}\left(p:q\right)=N_{\beta }\left(D\left(p:q\right),D\left(q:p\right)\right)$
skew (M,N)-*D* JS divergence	${\mathrm{JS}}_{D}^{M_{\alpha },N_{\beta }}\left(p:q\right):=N_{\beta }\left(D\left(p:{\left(pq\right)}_{\alpha }^{M}\right),D\left(q:{\left(pq\right)}_{\alpha }^{M}\right)\right)$

## Appendix B. Symbolic Calculations in Maxima

The program below calculates the normalizer *Z* for the harmonic *H*-mixtures of Cauchy distributions (Equation (133)).

assume(gamma>0);
Cauchy(x,gamma) := gamma/(%pi∗(x∗∗2+gamma∗∗2));
assume(alpha>0);
assume(alpha<1);
h(x,y,alpha) := (x∗y)/((1-alpha)∗y+alpha∗x);
assume(gamma1>0);
assume(gamma2>0);
m(x,alpha) := ratsimp(h(Cauchy(x,gamma1),Cauchy(x,gamma2),alpha));
/∗ calculate Z ∗/
integrate(m(x,alpha),x,-inf,inf);
